# Systemic and microcirculatory effects of blood transfusion in experimental hemorrhagic shock

**DOI:** 10.1186/s40635-017-0136-3

**Published:** 2017-04-21

**Authors:** Gonzalo Ferrara, Vanina S. Kanoore Edul, Héctor S. Canales, Enrique Martins, Carlos Canullán, Gastón Murias, Mario O. Pozo, Juan F. Caminos Eguillor, María G. Buscetti, Can Ince, Arnaldo Dubin

**Affiliations:** 1 Cátedra de Farmacología Aplicada, Facultad de Ciencias Médicas, Universidad Nacional de La Plata, La Plata, Argentina; 2 Translational Physiology, Academic Medical Center, University of Amsterdam, Amsterdam, Netherlands

**Keywords:** Hemorrhage, Shock, Transfusion, Microcirculation, Hypoxia

## Abstract

**Background:**

The microvascular reperfusion injury after retransfusion has not been completely characterized. Specifically, the question of heterogeneity among different microvascular beds needs to be addressed. In addition, the identification of anaerobic metabolism is elusive. The venoarterial PCO_2_ to arteriovenous oxygen content difference ratio (P_v-a_CO_2_/C_a-v_O_2_) might be a surrogate for respiratory quotient, but this has not been validated. Therefore, our goal was to characterize sublingual and intestinal (mucosal and serosal) microvascular injury after blood resuscitation in hemorrhagic shock and its relation with O_2_ and CO_2_ metabolism.

**Methods:**

Anesthetized and mechanically ventilated sheep were assigned to stepwise bleeding and blood retransfusion (*n* = 10) and sham (*n* = 7) groups. We performed analysis of expired gases, arterial and mixed venous blood gases, and intestinal and sublingual videomicroscopy.

**Results:**

In the bleeding group during the last step of hemorrhage, and compared to the sham group, there were decreases in oxygen consumption (3.7 [2.8–4.6] vs. 6.8 [5.8–8.0] mL min^−1^ kg^−1^, *P* < 0.001) and increases in respiratory quotient (0.96 [0.91–1.06] vs. 0.72 [0.69–0.77], *P* < 0.001). Retransfusion normalized these variables. The P_v-a_CO_2_/C_a-v_O_2_ increased in the last step of bleeding (2.4 [2.0–2.8] vs. 1.1 [1.0–1.3], *P* < 0.001) and remained elevated after retransfusion, compared to the sham group (1.8 [1.5–2.0] vs. 1.1 [0.9–1.3], *P* < 0.001). P_v-a_CO_2_/C_a-v_O_2_ had a weak correlation with respiratory quotient (Spearman *R* = 0.42, *P* < 0.001). All the intestinal and sublingual microcirculatory variables were affected during hemorrhage and improved after retransfusion. The recovery was only complete for intestinal red blood cell velocity and sublingual total and perfused vascular densities.

**Conclusions:**

Although there were some minor differences, intestinal and sublingual microcirculation behaved similarly. Therefore, sublingual mucosa might be an adequate window to track intestinal microvascular reperfusion injury. Additionally, P_v-a_CO_2_/C_a-v_O_2_ was poorly correlated with respiratory quotient, and its physiologic behavior was different. Thus, it might be a misleading surrogate for anaerobic metabolism.

**Electronic supplementary material:**

The online version of this article (doi:10.1186/s40635-017-0136-3) contains supplementary material, which is available to authorized users.

## Background

Hemorrhagic shock is a major cause of morbidity and mortality after trauma and other conditions. The restoration of systemic oxygen transport may not prevent the development of multiple organ failure. Possible explanations for this phenomenon are the oxygen debt accumulated during shock [1], the reperfusion injury mainly related to oxygen species production [2], and the persistent depression of microvascular perfusion [3]. Although the microcirculation in experimental hemorrhagic shock has been extensively described [4, 5], the microcirculatory alterations after blood resuscitation have not been completely studied. Specifically, the issue of perfusion heterogeneity among different microvascular beds needs to be adequately addressed.

The identification of anaerobic metabolism after the normalization of systemic hemodynamics in shock states is elusive. Even though several systemic variables may track the presence of tissue hypoxia, none of them is specific or sensitive. The acute increase in respiratory quotient (RQ) is an excellent marker of ongoing anaerobic metabolism in both exercise and oxygen supply dependency [6–8]. In both circumstances, there is an excess of CO_2_ production (VCO_2_) compared to oxygen consumption (VO_2_), which results from anaerobic VCO_2_. This arises from bicarbonate buffering of anaerobically generated protons (i.e., lactic acid dissociation, ATP hydrolysis). The measurement of RQ, however, requires analysis of expired gases by means of a metabolic cart. Such monitoring is usually not available in the ICU. Recently, observational studies found that venoarterial PCO_2_ difference (P_v-a_CO_2_) to arteriovenous oxygen content difference (C_a-v_O_2_) ratio might be a surrogate for RQ [9, 10]. This assumption relies on Fick’s principle, which states that VCO_2_ and VO_2_ can be calculated as the product of cardiac output by the respective venoarterial content difference. This also assumes a linear relationship between CO_2_ content and pressure. Nevertheless, those studies have not compared P_v-a_CO_2_/C_a-v_O_2_ with RQ.

Our goal was to characterize the intestinal mucosal and serosal microvascular alterations during hemorrhagic shock and retransfusion (H/R) and its relation with O_2_ and CO_2_ metabolism. A secondary objective was to correlate gut abnormalities with those of sublingual mucosa, a more accessible window in critically ill patients. Our hypotheses were (1) intestinal mucosal microcirculation is more susceptible than the other territories and (2) P_v-a_CO_2_/C_a-v_O_2_ fails to reflect the changes in RQ.

## Methods

### Anesthesia and ventilation

Seventeen sheep (23 ± 7 kg, mean ± SD) were anesthetized with 30 mg kg^−1^ of sodium pentobarbital and intubated and mechanically ventilated with a Servo Ventilator 900C (Siemens-Elema AB, Solna, Sweden) with a tidal volume of 15 mL kg^−1^, a FiO_2_ of 0.21, and a positive end-expiratory pressure of 6 cmH_2_O. The initial respiratory rate was set to keep the arterial PCO_2_ between 35 and 40 mmHg. This respiratory setting was maintained during the rest of the experiment. Neuromuscular blockade was performed with pancuronium bromide (0.06 mg kg^−1^). Additional pentobarbital boluses (1 mg kg^−1^) were administered hourly and when clinical signs of inadequate depth of anesthesia were evident. Analgesia was provided by fentanyl as a bolus of 2 μg kg^−1^, followed by 1 μg h^−1^ kg^−1^. These drugs were administered intravenously.

### Surgical preparation

A 7.5-French Swan-Ganz Standard Thermodilution Pulmonary Artery Catheter (Edwards Life Sciences, Irvine, CA, USA) was inserted through an introducer in the right external jugular vein to obtain mixed venous samples; its side port was used to administer fluids and drugs. Catheters were placed in the descending aorta via the left femoral artery to measure blood pressure, perform the bleeding, and obtain blood samples, and in the inferior vena cava to perform the retransfusion.

A midline laparotomy was performed, followed by a gastrostomy to drain gastric contents, and a splenectomy to avoid spleen contraction during the hemorrhage. An electromagnetic flow probe was placed around the superior mesenteric artery to measure blood flow (SMABF). A catheter was introduced in the mesenteric vein through a small vein proximal to the gut to draw blood samples and to measure pressure. A tonometer was inserted through a small ileotomy to measure intramucosal PCO_2_. A 10- to 15-cm segment of the ileum was mobilized, placed outside the abdomen, and opened 2 cm on the antimesenteric border to allow an examination of mucosal microcirculation. The exteriorized intestinal segment was covered and moisture and temperature preserved by a device. Finally, after complete hemostasis, the abdominal-wall incision was closed, excepting a short segment for externalization of the ileal loop.

### Measurements and derived calculations

Systemic VO_2_, VCO_2_, and RQ were measured by analysis of expired gases (MedGraphics CPX Ultima, Medical Graphics Corporation, St. Paul, MN). VO_2_ and VCO_2_ were adjusted to body weight.

Arterial, mixed venous, and mesenteric venous PO_2_, PCO_2_, pH, Hb, and O_2_ saturation were measured with a blood gas analyzer and a co-oximeter (ABL 5 and OSM 3, Radiometer, Copenhagen, Denmark). Oxygen-derived variables were calculated by standard formulae. Systemic and intestinal C_a-v_O_2_ were calculated using mixed and mesenteric venous O_2_ saturation and P_v-a_CO_2_/C_a-v_O_2_ by means of mixed venous blood.

Cardiac index (CI) was calculated as VO_2_ divided by systemic C_a-v_O_2_. Systemic oxygen transport (DO_2_) was calculated as CI by arterial O_2_ content.

SMABF was measured by the electromagnetic method (Spectramed Blood Flowmeter model SP 2202 B, Spectramed Inc., Oxnard, CA, USA), with in vitro calibrated transducers of 5–7 mm diameter (Blood Flowmeter Transducer, Spectramed Inc., Oxnard, CA, USA). Occlusive zero was controlled before and after each experiment. Non-occlusive zero was corrected before each measurement. SMABF was referred to gut weight. Intestinal DO_2_ was calculated as SMABF by arterial O_2_ content and intestinal VO_2_ as SMABF by intestinal C_a-v_O_2_.

Intramucosal PCO_2_ was measured by air tonometry (Tonometrics Catheter and Tonocap, Datex-Ohmeda, Helsinki, Finland). Then, we calculated intramucosal-arterial PCO_2_ (ΔPCO_2_).

Arterial lactate was measured with a point-of-care analyzer (Stat Profile Critical Care Xpress, Nova Biomedical, Waltham, MA, USA).

### Microvideoscopic measurements and analysis

The microcirculatory network was evaluated in intestinal mucosa and serosa, and sublingual mucosa by means of a sidestream-dark-field (SDF) imaging device (Microscan, MicroVision Medical, Amsterdam, Netherlands) [11]. Different precautions were taken and steps followed to obtain images of adequate quality and to insure satisfactory reproducibility. After gentle removal of saliva by isotonic-saline-drenched gauze, steady images of at least 20 s were obtained while avoiding pressure artifacts with a portable computer and an analog-to-digital video converter (ADVC110, Canopus Co., San Jose, CA, USA). The videos were recorded from three different areas. Video clips were stored as AVI files to allow computerized frame-by-frame image analysis.

Video-image analysis was performed blindly by well-trained researchers. Adequate focus and contrast adjustment were verified, and images of poor quality were discarded. The entire sequence was used to describe the semiquantitative characteristics of the microvascular flow and, particularly, the presence of stopped or intermittent flow.

We used an image-analysis software (Microscan analysis software®–AVA 3.0–MicroVision Medical, Amsterdam, Netherlands) [12] to determine total vascular density. An analysis based on semiquantitative criteria that distinguished no flow (0), intermittent flow [1], sluggish flow [2], and continuous flow [3] was performed on individual vessels [3]. The overall score, called microvascular flow index (MFI), is the average of the individual values [13]. Quantitative red blood cell (RBC) velocity was determined using space-time diagrams [12]. We also calculated the proportion of perfused vessels, the perfused vascular density (i.e., the total vascular density multiplied by the fraction of perfused vessels), and the heterogeneity flow index as highest-lowest MFI divided mean MFI [14].

In sheep, most of sublingual vascular density (97 ± 1%) and all intestinal vessels consist of small vessels (diameter <25 μm) [5], so analysis was focused on these types of vessels, whereas the vessels of higher diameter were assessed only for ruling out compression artifacts.

### Experimental procedure

Basal measurements were taken after a period of no less than 30 min after systemic VO_2_, VCO_2_, and SMABF became stable. Animals were then assigned to H/R (*n* = 10) or sham (*n* = 7) group. In the H/R group, three consecutive bleedings of 5–10 mL kg^−1^ were performed at 30-min intervals, until reaching reductions in systemic VO_2_ and increases in RQ. Then, shed blood was rapidly reinfused (~2 min) and sheep were followed during one additional hour. In the sham group, the same experimental preparation was carried out and 0.9% NaCl was infused to maintain hemodynamic variables at basal values, without further interventions. Measurements were performed at baseline (0′), during bleeding (30′, 60′, and 90′), and after retransfusion (2′, 30′, and 60′). Microcirculatory videos were only acquired at 0′, 30′, and 90′ of hemorrhage and 60′ of retransfusion, but in sublingual mucosa, images were also continuously obtained during blood reinfusion. Thereafter, the initial and the final portion of these videos (0′ and 2′) were analyzed. Blood temperature was kept constant throughout the study with a heating lamp.

At the end of the experiment, animals were killed with an additional dose of pentobarbital and a KCl bolus. A catheter was inserted in the superior mesenteric artery, and Indian ink was instilled through it. Dyed intestinal segments were dissected, washed, and weighed to calculate gut indexes.

### Data analysis

Because of the small numbers of animals, nonparametric tests were used. Changes over time within each group were assessed with nonparametric analysis of variance for repeated measurements (Friedman test) followed by a post hoc test (Dunn’s multiple comparison test). Differences between groups at each time point were analyzed with Mann-Whitney *U* test. Correlations between variables were calculated with Spearman test. Data are expressed as median and interquartile range. A *P* value <0.05 was considered statistically significant.

## Results

### Effects on systemic and intestinal hemodynamics and oxygen transport

**Fig. 1:**
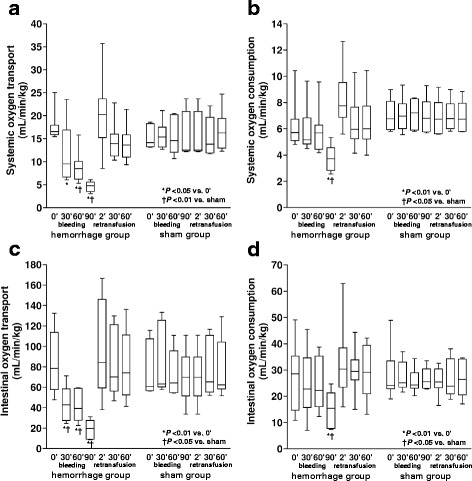
Behavior of systemic and intestinal O_2_ transport and consumption. **a** Systemic O_2_ transport. **b** Systemic O_2_ consumption. **c** Intestinal O_2_ transport. **d** Intestinal O_2_ consumption

**Fig. 2:**
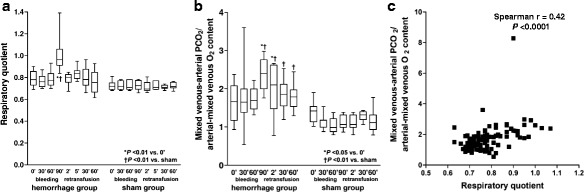
Behavior of the respiratory quotient and the venoarterial PCO_2_ to arteriovenous oxygen content difference ratio (P_v-a_CO_2_/C_a-v_O_2_). **a** Respiratory quotient. **b** P_v-a_CO_2_/C_a-v_O_2_. **c** Correlation between respiratory quotient and P_v-a_CO_2_/C_a-v_O_2_

**Fig. 3:**
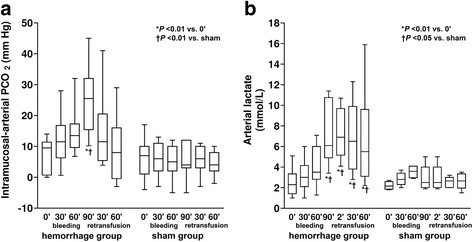
Behavior of intramucosal-arterial PCO_2_ difference and arterial lactate. **a** Intramucosal-arterial PCO_2_ difference. **b** Arterial lactate

In the H/R group, mean arterial pressure decreased from the first step of bleeding, while reductions in CI and SMABF reached statistical significance during the last stage (Table 1). Each variable was normalized after retransfusion, but at 2′, CI and SMABF were higher than baseline. During bleeding, there were progressive reductions in systemic and intestinal DO_2_ which, in the last step, were associated with systemic and intestinal VO_2_ falls and RQ increases (Figs. 1 and 2). During retransfusion, all these variables were normalized. Bleeding induced lactic acidosis, which persisted after retransfusion (Fig. 3).

**Table 1:** Hemoglobin, systemic and intestinal hemodynamic and oxygen transport variables, and arterial blood gases in hemorrhage/retransfusion (H/R) and sham groups

			Hemorrhage			Retransfusion		
		Baseline	30′	60′	90′	2′	30′	60′
Hemoglobin (g per 100 mL)	H/R	8.3 [7.7–9.7]	7.8 [7.3–9.1]†	7.3 [7.0–9.1]*†	6.5 [6.1–7.7]*†	8.0 [6.9–9.3]†	7.8 [7.7–9.7]	8.1 [7.2–9.7]
	Sham	9.9 [9.3–10.3]	9.9 [9.5–10.4]	9.8 [9.3–10.5]	9.6 [9.2–9.8]	9.6 [9.2–9.8]	9.3 [8.8–9.8]	9.4 [8.6–10.0]
Mean arterial pressure (mmHg)	H/R	83 [72–96]	42 [34–47]*†	40 [32–44]*†	28 [22–30]*†	95 [90–100]	90 [82–99]	91 [79–97]
	Sham	91 [90–102]	97 [80–109]	92 [89–102]	99 [91–109]	98 [90–108]	100 [92–112]	100 [91–110]
Central venous pressure (mmHg)	H/R	3 [0–6]	3 [1–6]	4 [1–7]	4 [0–7]	6 [1–9]	3 [1–5]	3 [0–5]
	Sham	2 [1–2]	2 [1–4]	3 [1–3]	2 [1–4]	2 [1–4]	2 [1–4]	2 [1–4]
Mean pulmonary pressure (mmHg)	H/R	17 [10–20]	12 [7–17]	11 [8–13]	10 [6–12]*†	22 [17–32]†	18 [12–20]	16 [13–19]
	Sham	11 [10–13]	13 [12–14]	14 [13–15]	13 [12–13]	13 [12–13]	14 [13–15]	13 [11–16]
Pulmonary occlusion pressure (mmHg)	H/R	5 [4–7]	6 [1–8]	4 [1–8]	6 [0–7]	7 [4–9]	5 [2–9]	4 [2–7]
	Sham	3 [2–6]	2 [2–4]	2 [2–3]	3 [3–4]	3 [3–4]	2 [1–4]	2 [2–3]
Cardiac index (mL min^−1^ kg^−1^)	H/R	155 [136–177]	89 [64–177]	78 [70–119]	53 [39–72]*†	210 [158–340]†	133 [116–179]	134 [102–180]
	Sham	122 [101–149]	122 [100–151]	108 [97–167]	99 [94–168]	101 [96–112]	110 [99–178]	113 [96–174]
Mesenteric flow (mL min^−1^ per 100 g)	H/R	805 [489–903]	451 [253–665]	427 [264–576]	236 [110–332]*†	1052 [644–1406]†	850 [550–1072]	923 [523–986]
	Sham	457 [445–786]	500 [457–992]	445 [426–743]	457 [379–714]	448 [428–750]	487 [457–892]	502 [487–814]
Arterial pH	H/R	7.44 [7.35–7.49]	7.44 [7.39–7.49]	7.44 [7.40–7.49]	7.42 [7.39–7.48]	7.28 [7.20–7.37]*†	7.37 [7.29–7.43]†	7.41 [7.32–7.45]†
	Sham	7.49 [7.42–7.51]	7.49 [7.39–7.56]	7.52 [7.41–7.52]	7.53 [7.42–54]	7.52 [7.41–7.53]	7.51 [7.44–55]	7.55 [7.42–54]
Arterial PCO_2_ (mmHg)	H/R	38 [37–40]	36 [35–39]	36 [33–39]	34 [31–36]*	44 [41–52]*†	38 [36–42]	39 [34–41]
	Sham	38 [33–40]	37 [35–40]	36 [34–39]	34 [33–39]	34 [33–39]	34 [32–38]	34 [32–38]
Arterial PO_2_ (mmHg)	H/R	86 [77–91]	83 [77–93]	88 [77–95]	92 [87–106]	75 [69–84]*†	86 [75–92]	85 [77–94]
	Sham	87 [78–93]	83 [81–87]	87 [83–95]	87 [78–99]	91 [74–99]	88 [87–100]	89 [85–99]
Arterial bicarbonate (mEq L^−1^)	H/R	26 [21–28]	25 [22–27]	23 [20–26]*	23 [18–25]*†	23 [18–25]*†	23 [19–25]*†	24 [19–27]†
	Sham	26 [25–28]	29 [23–31]	28 [25–29]	27 [25–29]	27 [25–29]	27 [25–28]	28 [25–30]
Arterial base excess (mEq L^−1^)	H/R	2 [−4 to 4]	1 [−2 to 5]	2 [−2 to 3]	−1 [−5 to 3]*†	−3 [−9 to 0]*†	−2 [−6 to 1]*†	−1 [−6 to 2]†
	Sham	3 [−3 to 5]	5 [−2 to 9]	5 [1–6]	4 [0–6]	4 [0–6]	4 [0–5]	5 [1–8]

Data are shown as median [percentile 0.25–0.75]

*P < 0.05 vs. basal; †*P* < 0.05 vs. sham

### Effects on CO_2_ metabolism

Compared to baseline, VCO_2_ decreased in the last step of bleeding and increased at 2′ of retransfusion. Systemic and intestinal P_v-a_CO_2_ and ΔPCO_2_ augmented during bleeding and were normalized during retransfusion (Fig. 2 and Table 2). ΔPCO_2_ correlated with intestinal mucosal total vascular density (*R* = −0.44, *P* = 0.0002), perfused vascular density (*R* = −0.43, *P* = 0.0003), proportion of perfused vessels (*R* = −0.48, *P* < 0.0001), RBC velocity (*R* = −0.35, *P* < 0.001), MFI (*R* = −0.52, *P* < 0.0001), and heterogeneity flow index (*R* = 0.52, *P* < 0.0001).

**Table 2:** Systemic CO_2_ production and venoarterial PCO_2_ differences in hemorrhage/retransfusion (H/R) and sham groups

			Hemorrhage			Retransfusion		
		Baseline	30′	60′	90′	2′	30′	60′
CO_2_ production (mL min^−1^ kg^−1^)	H/R	4.5 [4.2–5.0]	4.1 [3.6–5.3]	4.3 [3.8–4.8]	3.6 [3.0–4.4]*†	6.2 [5.0–7.5]*†	4.7 [4.2–6.4]	4.7 [4.0–5.3]
	Sham	4.9 [4.5–5.4]	4.8 [4.7–5.8]	4.9 [4.4–6.0]	4.8 [4.5–5.3]	4.8 [4.5–5.4]	4.8 [4.3–5.8]	4.5 [4.3–5.9]
Mixed venoarterial PCO_2_ (mmHg)	H/R	6 [5–7]†	10 [7–12]*	11 [9–12]*†	16 [14–20]*†	9 [4–12]	8 [6–10]	8 [7–10]
	Sham	8 [7–10]	7 [6–8]	6 [6–8]	6 [6–8]	7 [6–8]	7 [6–8]	6 [5–7]
Mesenteric venoarterial PCO_2_ (mmHg)	H/R	5 [5–6]	8 [8–10]*†	11 [10–12]*†	15 [12–22]*†	6 [4–13]	6 [5–11]	6 [5–7]
	Sham	6 [4–12]	5 [4–6]	6 [5–7]	6 [5–7]	6 [5–7]	6 [4–9]	5 [4–7]

Data are shown as median [percentile 0.25–0.75]

*P < 0.05 vs. basal; †*P* < 0.05 vs. sham

P_v-a_CO_2_/C_a-v_O_2_ increased during bleeding and, during retransfusion, remained higher than that of the sham group. This ratio correlated with RQ (*R* = 0.42, *P* < 0.0001) (Fig. 2).

### Effects on microcirculation

From the first step of bleeding, each intestinal and sublingual microcirculatory variable was compromised.

During retransfusion, all the variables improved in the three territories. However, proportion of perfused vessels, microvascular flow index, and heterogeneity flow index could not be normalized. RBC velocity returned to basal values in intestinal mucosa and serosa and persisted diminished in sublingual mucosa. Conversely, total and perfused vascular density remained low in intestinal mucosa and serosa and were restored to baseline in sublingual mucosa.

**Fig. 4:**
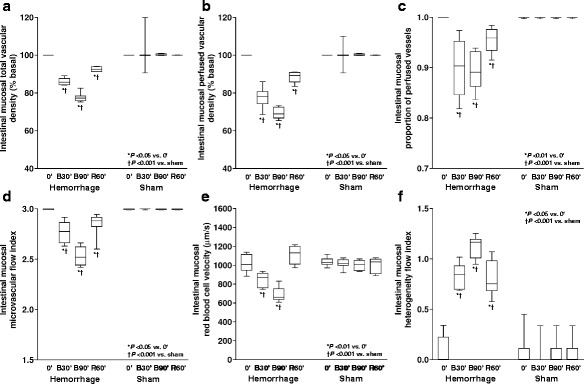
Behavior of the intestinal mucosal microcirculatory variables. **a** Total vascular density. **b** Perfused vascular density. **c** Proportion of perfused vessels. **d** Microvascular flow index. **e** Red blood cell velocity. **f** Heterogeneity flow index

**Fig. 5:**
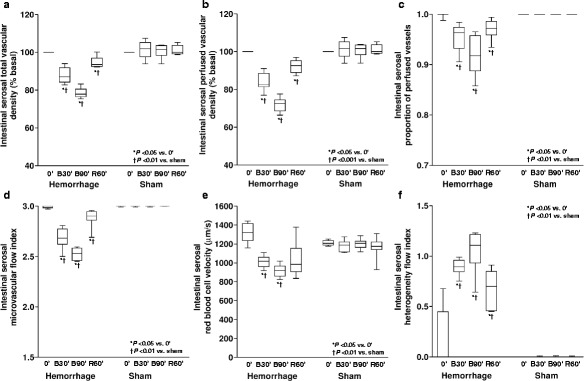
Behavior of the intestinal serosal microcirculatory variables. **a** Total vascular density. **b** Perfused vascular density. **c** Proportion of perfused vessels. **d** Microvascular flow index. **e** Red blood cell velocity. **f** Heterogeneity flow index

**Fig. 6:**
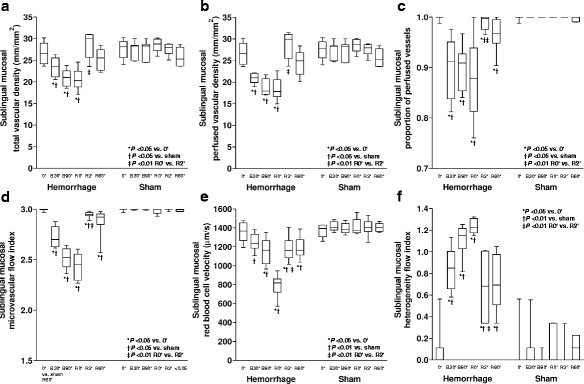
Behavior of the sublingual microcirculatory variables. **a** Total vascular density. **b** Perfused vascular density. **c** Proportion of perfused vessels. **d** Microvascular flow index. **e** Red blood cell velocity. **f** Heterogeneity flow index



In sublingual mucosa, each microvascular variable improved after 2′ of blood reinfusion. Such variables were similar at 2′ and 60′ of retransfusion (Figs. 4, 5, and 6 and the video in Additional file 1).

**Additional file 1:** Video sublingual microcirculation during retransfusion. The video was continuously acquired during the period of shed blood reinfusion. Segments of the whole video were cut and edited. The left lower corner shows the actual time. There were sharp and fast increases in flow velocity and density. (WMV 13046 kb)


## Discussion

Our main finding was that retransfusion improved the microcirculatory alterations that developed in hemorrhagic shock. However, subtle abnormalities persisted in the face of the normalization of aerobic metabolism. Microvascular reperfusion injury was present in the three studied vascular beds, with minor differences among them. In addition, P_v-a_CO_2_/C_a-v_O_2_ only showed a weak correlation with RQ and, mainly, remained elevated during retransfusion.

In experimental hemorrhagic shock, the reports of the microcirculatory effects of blood transfusion are controversial. For example, in rats, transfusion deteriorated perfused capillary density of gastric mucosa [15]. Conversely, in another study, RBC administration restored conjunctival microcirculation and muscle tissue oxygenation [16]. In the hamster window chamber model, packed fresh RBC improved but not completely normalized functional capillary density and flow [17, 18]. Partial beneficial effects were also found on rat hepatic and ileum microcirculation [19, 20].

These inconsistent results might be related to differences in the studied species, in the severity of shock, and in the particular microvascular bed evaluated. Accordingly, it has been suggested that the gut might be less responsive to blood resuscitation than the heart [21]. We found beneficial effects of blood resuscitation on intestinal and sublingual microcirculation. The recovery of microvascular perfusion, however, was incomplete, and regional deficits persisted. The novelty of our study consists in the assessment of different and relevant microvascular beds, along with a comprehensive evaluation of systemic and regional hemodynamics and oxygenation.

Microcirculatory reperfusion injury exhibited different manifestations in sublingual and intestinal territories. Although in the three areas blood reinfusion improved all the microvascular variables, only sublingual densities and intestinal RBC velocity were completely normalized. Taking into account these minor regional differences, the sublingual mucosa might be an adequate window for the monitoring of reperfusion microvascular injury. This situation might differ in other forms of distributive shock. After the resuscitation of septic shock, microcirculatory derangements are more severe in intestinal than in sublingual mucosa [22–24]. In a sheep model of endotoxic shock, the hemodynamic normalization by means of fluids corrected sublingual microcirculation whereas intestinal villi remained hypoperfused [22]. The reasons for this higher heterogeneity between microvascular beds in sepsis than in our model reperfusion injury are uncertain but might be related to different mechanisms of damage as well as the modality of resuscitation (colloid or crystalloid solutions vs. blood).

The subtle villi abnormalities could trigger mechanisms of tissue damage such as the alteration in the mucosa barrier dysfunction and systemic translocation of bacteria and their products [25]. What is more, in patients with traumatic hemorrhagic shock, the persistence of microcirculatory alterations after resuscitation predicted the development of multiorgan failure [26]. The microcirculatory dysfunction has also been proposed as the link between trauma and coagulopathy [27]. In addition, the presence of sublingual microvascular alterations in trauma patients might help to select patients who will benefit from blood transfusion. In patients with hemorrhagic shock, blood transfusion improved sublingual microcirculation independently of macrocirculation and hemoglobin level. The change in microvascular perfusion, however, was negatively correlated with the basal microvascular perfusion [28]. A similar response was described in trauma patients with hemodynamic stability [29]. The identification of microcirculatory disorders might thus contribute to the evaluation of patients with hemorrhagic shock.

Another original approach of this study was the continuous video acquisition in the sublingual area during retransfusion. This allowed a thorough assessment of the timing of capillary recruitment. While many microvascular parameters did not reach basal values, all microcirculatory variables improved sharply in the 2-min period of observation. In addition, the values at 2′ were similar to those observed at the end of the reperfusion phase, evidencing that the changes in systemic hemodynamics rapidly recruited the microcirculation. Hence, we found a coherence between macro- and microcirculation, which was only partial, since most of the microvascular variables stayed altered.

The ΔPCO_2_ is considered a sensitive marker of mucosal perfusion [22]. This study confirms the dependency of ΔPCO_2_ on microcirculation. Nevertheless, ΔPCO_2_ normalized at the end of the retransfusion period when microcirculatory alterations were still present. Consequently, our results suggest that tissue capnometry is less sensitive than videomicroscopy to disclose the presence of mucosal hypoperfusion.

Some studies have suggested that P_v-a_CO_2_/C_a-v_O_2_ could be an adequate surrogate for RQ [9, 10]. Moreover, a cutoff of 1.4 might point out the presence of anaerobic metabolism. Nevertheless, P_v-a_CO_2_/C_a-v_O_2_ has never been compared to RQ. Our results showed that the correlation between both variables was weak, and more importantly, the physiological behavior was different. Given that RQ normalized after retransfusion, the increased P_v-a_CO_2_/C_a-v_O_2_ should be ascribed to increased release of CO_2_ from hemoglobin, not to tissue hypoxia. The persistent P_v-a_CO_2_/C_a-v_O_2_ elevation might result from changes in venous oxygen saturation (Haldane effect) and hemoglobin levels and mostly from persistent hyperlactatemia [30, 31]. All these factors can shift the CO_2_Hb dissociation curve. Since P_v-a_CO_2_/C_a-v_O_2_ might be a misleading indicator of RQ and anaerobic metabolism, its values should be carefully interpreted. This is additionally emphasized by the fact that ongoing anaerobic metabolism is identified by acute increases in RQ, not by isolated values [6–8]. Actually, the normal range of RQ is 0.67 to 1.30 [32].

Our study has some limitations. First, capillary reperfusion failure has been attributed to several mechanisms, including microthrombosis, leukocyte plugging, endothelial cell swelling, vasomotor dysfunction, and capillary narrowing due to edema [3]. Our study was only descriptive and did not address such mechanisms. In addition, we only studied three microvascular territories and the microcirculation might have behaved differently in other vascular beds. Finally, the assessment of retransfusion was limited to 60 min. Longer observation periods might have produced different results.

## Conclusions

Our main findings were that reperfusion microvascular injury developed in intestinal and sublingual areas, despite the complete restoration of aerobic metabolism. Since differences among microvascular beds were minor, sublingual mucosa might be an adequate window for the monitoring of intestinal reperfusion injury. Finally, given that P_v-a_CO_2_/C_a-v_O_2_ only had a poor correlation with RQ and a different physiologic behavior, it seems to be an inadequate surrogate for RQ.

## Abbreviations

C_a-v_O_2_: Arteriovenous oxygen content difference
CI: Cardiac index
DO_2_: Oxygen transport
H/R: Hemorrhagic shock and retransfusion
MFI: Microvascular flow index
P_v-a_CO_2_: Venoarterial PCO_2_ difference
P_v-a_CO_2_/C_a-v_O_2_: Ratio of venoarterial PCO_2_ to arteriovenous oxygen content difference
RBC: Red blood cell
RQ: Respiratory quotient
SDF: Sidestream dark field
SMABF: Superior mesenteric artery blood flow
VCO_2_: Carbon dioxide production
VO_2_: Oxygen consumption
ΔPCO_2_: Intramucosal-arterial PCO_2_
